# Mechanistic Roles of Noncoding RNAs in Lung Cancer Biology and Their Clinical Implications

**DOI:** 10.1155/2012/737416

**Published:** 2012-07-18

**Authors:** Katey S. S. Enfield, Larissa A. Pikor, Victor D. Martinez, Wan L. Lam

**Affiliations:** ^1^British Columbia Cancer Research Center, Vancouver, BC, Canada V5Z 1L3; ^2^Interdisciplinary Oncology Program, University of British Columbia, Vancouver, BC, Canada V5Z1L3; ^3^Department of Pathology and Laboratory Medicine, University of British Columbia, Vancouver, BC, Canada V6T2B5

## Abstract

Lung cancer biology has traditionally focused on genomic and epigenomic deregulation of protein-coding genes to identify oncogenes and tumor suppressors diagnostic and therapeutic targets. Another important layer of cancer biology has emerged in the form of noncoding RNAs (ncRNAs), which are major regulators of key cellular processes such as proliferation, RNA splicing, gene regulation, and apoptosis. In the past decade, microRNAs (miRNAs) have moved to the forefront of ncRNA cancer research, while the role of long noncoding RNAs (lncRNAs) is emerging. Here we review the mechanisms by which miRNAs and lncRNAs are deregulated in lung cancer, the technologies that can be applied to detect such alterations, and the clinical potential of these RNA species. An improved comprehension of lung cancer biology will come through the understanding of the interplay between deregulation of non-coding RNAs, the protein-coding genes they regulate, and how these interactions influence cellular networks and signalling pathways.

## 1. Introduction

The human genome is comprised of less than 2% protein coding genes; however, more than 90% of the genome is transcribed, suggesting that the majority of the transcriptome is comprised of noncoding RNAs—transcripts that lack an open reading frame and as such do not encode a protein [1–4]. However this by no mean implies that ncRNAs lack function, but rather highlights the importance of looking beyond protein-coding genes in order to improve our knowledge of normal and disease biology. ncRNAs are loosely classified into two main categories: small non-coding RNAs (18–200 nucleotides), which includes transcripts such as miRNAs, transfer RNAs (tRNAs), small interfering RNAs (siRNAs), piwi-interacting RNAs (piRNAs) and some ribosomal RNAs, and long non-coding RNAs (lncRNAs) (200+ nucleotides), a family comprised of pseudogenes, antisense RNA and transcribed ultraconserved regions to name a few (Table 1) [4]. ncRNAs comprise a class of transcripts that until the last few decades was largely overlooked. While some are known to play important roles in the regulation of gene expression, splicing, epigenetic control, chromatin structure and nuclear transport, the function of most ncRNAs remains unknown [5, 6]. Of the species of ncRNAs identified to date, miRNAs, siRNAs, and piRNAs are the most thoroughly investigated. With roles in a number of cellular functions, it is not surprising that the deregulation of ncRNAs has been linked to human disease, including a number of cancers, such as breast, prostate, lung, colon, and liver. Increasing evidence that ncRNAs, beyond miRNAs, may be primary genetic regulators has led to the hypothesis that they may be ideal diagnostic markers and therapeutic targets [4].

Lung cancer is the leading cause of cancer deaths worldwide. The consistent poor 5-year survival rate of 15%, owing largely to the late stage of diagnosis and a lack of effective therapeutics, underscores the need for novel therapeutic modalities as well as early detection and prognostic markers [7–9]. While protein coding genes remain the primary focus of current genomic and proteomic studies, deregulation of ncRNAs has a demonstrated role in the regulation of gene expression and warrants continued investigation. Clinically, ncRNAs are emerging as potential tools and targets in lung cancer. miRNA expression profiles have been associated with lung cancer prognosis, disease progression, survival, and outcome prediction as well as discrimination of subtypes [10–13]. In fact, a recent study deemed miRNA expression signatures superior to global mRNA expression profiles in the accurate classification of NSCLC subtypes [14]. While lncRNAs in lung cancer is still an emerging field, several have been shown to be involved in tumorigenesis, including *HOTAIR, H19, ANRIL, *and *MALAT1* [4, 15]. Other ncRNAs implicated in lung cancer include microRNA offset RNAs (moRNA) and *piRNA-651* [16, 17]. While new ncRNAs important to lung cancer continue to be discovered, miRNAs and lncRNAs constitute the majority of known cancer related non-coding transcripts. This paper therefore focuses on miRNAs and lncRNAs; their mechanisms of disruption, current technologies for detection and analysis, their role in lung cancer, and their impact on lung cancer diagnosis and treatment.

## 2. miRNAs and Their Role in Lung Cancer

miRNAs are small non-coding RNAs approximately 18–25 nucleotides in length that negatively regulate gene expression posttranscriptionally [18]. miRNAs have been shown to regulate a number of critical biological processes, including but not limited to, proliferation, apoptosis, metabolism, epithelial to mesenchymal transition, differentiation, and cellular development, acting as both oncogenes and tumor suppressors [14, 18]. miRNAs are transcribed by RNA polymerase II (pol II) into long, double-stranded stem-loop containing primary (pri)-miRNAs, typically hundreds-to-thousands of nucleotides in length. The pri-miRNA is processed into a shorter double-strandeded RNA of 70 nucleotides (pre-miRNA) by the endonuclease Drosha, exported to the cytoplasm via XPO5, and further processed to a length of 22 nucleotides (mature duplex) by the endonucleases and Dicer. Dissociation of the miRNA double strand duplex and incorporation of the mature strand into the RNA-induced silencing complex (RISC) guides RISC to the target mRNA, where the miRNA targets the 3′ UTR, or less frequently the 5′ UTR [19], of the mRNA based on sequence similarity. Translation of the mRNA is ultimately prevented either by transcript degradation, inhibition of translation, or mRNA decay, and depends on sequence complementarity between the miRNA and its mRNA target, the particular Ago protein in the RISC, and possibly the position and number of complementary nucleotides [20, 21]. Perfect complementarity leads to Ago2-mediated mRNA cleavage [22], while imperfect complementarity can lead either to transcript decay or translational inhibition via either Ago1, Ago3, or Ago4 [14, 23–25].

To date, over 1400 human miRNAs have been identified [26]. A single miRNA is capable of affecting multiple protein coding genes, while similarly a gene can be targeted by more than one miRNA. It is believed that over one-third of the genome is regulated by at least a single miRNA [24]. Frequently located at chromosomal breakpoints, fragile sites, regions of LOH or amplification, miRNAs are highly susceptible to genomic alterations and subsequently deregulated expression [27, 28]. Changes in miRNA expression have been detected in a variety of malignancies as well as preinvasive cancer and have been associated with clinical features such as prognosis and survival. As such, many miRNAs are currently under investigation as diagnostic and prognostic biomarkers, therapeutic targets, and as markers of disease subtypes [29].

The pathogenesis of lung cancer has been associated with the deregulation of several miRNAs (Table 2), altering cellular processes including angiogenesis, cell differentiation, invasion, and metastasis. *Let-7*, the first miRNA identified to be aberrantly expressed in lung cancer, targets *KRAS* and *HMGA2*, resulting in suppression of proliferation, with reduced *let-7* expression correlating with poor clinical outcome [12, 30]. Garofalo et al. showed that overexpression of *miR-221* and -*222* enhances cellular migration through activation of AKT, impairs TRAIL-dependent apoptosis by targeting *PTEN* and correlates with aggressive nonsmall cell lung cancer (NSCLC) [31]. Similarly, the downregulation of the *miR-34* family leads to increased proliferation and inhibition of apoptosis through the p53 pathway and clinically correlates with a higher risk of relapse [32]. Studies in lung cancer cell lines have also revealed a number of important miRNAs, including *miR-125a*, -*126,* and -*206*, the overexpression of which have all been shown to be associated with invasive and metastatic potential [33, 34]. While miRNAs are now appreciated as key regulators of gene expression in lung cancer, capable of classifying histological subtypes, and predicting recurrence, progression, and prognosis, they are not the only class of ncRNA implicated in lung tumorigenesis [13, 35].

## 3. LncRNAs: Emerging Players in Lung Cancer

LncRNAs are largely polyadenylated RNAs greater than 200 nucleotides in length that regulate gene expression through epigenetic regulation, splicing, imprinting, transcriptional regulation and subcellular transport [5, 6, 36, 37]. Although originally regarded as transcriptional noise, lncRNAs function in both *cis*, such as antisense non-coding* RNA *in the* INK4 locus* (*ANRIL*) which complexes with Polycomb Repressive Complex 2 (PRC2) to act on the same chromosome [38], and trans, such as *HOX *antisense intergenic* RNA* (*HOTAIR*) whose association with PRC2 affects different chromosomes [39]. LncRNAs demonstrate developmental stage and tissue specificity, indicating their expression is tightly regulated [5, 40–44]. They are loosely categorized by their position relative to coding genes as intergenic, intragenic/intronic and antisense [40]. Current estimates of lncRNA content range from 7000–23,000 unique lncRNAs, with a growing cohort being validated as having a role in human disease processes [42, 45]. Included in this list are a number of human cancers, suggesting that aberrant expression of lncRNAs contributes to tumorigenesis, and highlighting the need to better understand the mechanisms through which these transcripts exert their function.

The first lncRNAs identified were the imprinted *H19* gene and *X-inactive-specific transcript (XIST)* critical to X chromosome inactivation, although at the time of discovery they were not coined “lncRNAs.” Since then, lncRNAs have been associated with Alzheimers, Fragile X Syndrome, blepharophimosis syndrome (BPES), and cancer [46–48]. *HOTAIR*, located in the *HOXC* locus on 12q13.13, was one of the first lncRNAs to be described as having a fundamental role in cancer [49]. *HOTAIR* associates with PRC2, silencing a portion of the *HOXD* locus and inducing H3 lysine 27 trimethylation, subsequently remodelling the gene expression pattern of breast epithelial cells to more closely resemble embryonic fibroblasts [39, 49]. *HOTAIR* is overexpressed in roughly one quarter of human breast cancers and was found to be an independent prognostic marker of poor survival and metastasis as well as a driver of metastasis in murine models [39]. At this time, a handful of lncRNAs have been associated with lung cancer (Table 3), most significantly metastasis-associated lung adenocarcinoma transcript 1 (*MALAT1*), a lncRNA that is associated with high metastatic potential and poor patient prognosis in NSCLC [15]. *MALAT1* is upregulated in a variety of other human cancers including breast, colon, prostate, and liver cancer. Its exact mechanism of action is under investigation, although it is thought to regulate the level of phosphorylated splicing factors, thereby affecting alternative splicing in the cell [50].

## 4. Mechanisms of miRNA and LncRNA Disruption in Lung Cancer

The identification of genetic and epigenetic mechanisms of ncRNA deregulation provides insight into which ncRNAs are aberrantly expressed and therefore relevant to lung cancer biology. Similar to protein coding genes, ncRNAs can be deregulated by multiple genetic and epigenetic mechanisms. Deregulation occurs both directly at the miRNA or lncRNA loci, and indirectly through disruption of processing components or alterations to target transcripts. As miRNA deregulation has been studied more comprehensively than lncRNAs, it will be discussed in greater detail.

### 4.1. Copy Number Alterations

miRNA loci are often located at regions of genomic instability and as such are highly susceptible to genomic alterations [28]. Perhaps the most well-described example of direct alteration to a miRNA is the loss of *let-7* [30, 51–54]. Acting as tumor suppressive miRNAs, the *let-7* family members are located in chromosomal regions frequently deleted in lung and other cancers, including 3p, 9q, and 21p. A well-characterized example of oncogenic miRNAs is the *miR-17-92* cluster, amplified in small cell lung cancer (SCLC) tumors and cell lines [55]. Expression of this cluster has been shown to be regulated by MYC, an oncogene frequently overexpressed in lung cancer [51]. Conversely, gene dosage alterations affecting lncRNA expression have yet to be reported.

### 4.2. Epigenetic Mechanisms

Epigenetic influences, including the effects of global hypomethylation and site-specific hypermethylation found in cancer genomes, have been investigated with reference to miRNAs. Diederichs and Haber showed that treatment of the A549 cell line with demethylating agent 5-azacytidine did not result in significantly altered miRNA expression by microarray analysis [56]. However, increasing evidence suggests that miRNAs are subject to epigenetic regulation. For example, the promoter region of *miR-34a* is known to be hypermethylated in lung and other cancer cell lines [57], while *let-7a-3* has been found to be hypomethylated in lung adenocarcinoma compared to normal lung tissue [58]. Downregulation of *miR-1*, can be reversed by histone deacetylase activity, suggesting its and possibly other miRNAs deregulation occurs via histone modification [59]. Indirect alterations to DNA methylation patterns can also occur through miRNA targeting of DNA methyltransferases. For example, *miR-29 a, b,* and *c* were shown to directly target both *DNMT3A* and *DNMT3B*, two genes that are often overexpressed in lung cancer [60], resulting in aberrant DNA methylation.

### 4.3. Single-Nucleotide Polymorphisms

Unlike protein-coding genes, SNPs within the functional seed sequences of miRNAs are rare, occurring in <1% of miRNAs [61]. Exact sequence matches observed in specific regions of *let-7* among different species demonstrate that miRNAs have evolutionarily conserved functions [62] and demonstrate the presence of negative selection against sequence variations. However, in lung cancer, SNPs have been identified within pri- and pre-miRNA sequences [63–67], within or near miRNA binding sites [68], and within genes encoding miRNA processing machinery [69–72]. A SNP located in pre-miRNA region of miR-196a2 (rs11614913 homozygous variant) was associated with significantly increased risk and poor survival among Chinese lung cancer patients [65]. Additionally, a G-to-T variant (rs3134615) in the 3′ UTR of *L-MYC* can inhibit the interaction between *miR-1827 *and the *L-MYC* target region, resulting in a constitutively higher expression level of *L-MYC* and an increased risk of SCLC in the Chinese population studied [73]. Furthermore, a SNP in the 3′ UTR of *KRAS* was able to alter its *let-7*-mediated regulation and was linked to increased risk of NSCLC among moderate smokers [74]. Interestingly, some of these SNPs seem to be lung cancer-specific. For example, while individuals carrying the CC genotype of the rs11614913 hsa-mir-196a2 variant have increased susceptibility to lung cancer, this relationship was not observed in hepatocellular carcinoma, gastric, or esophageal cancer patients [65, 74–76]. Finally, some SNPs relating to miRNA function can potentially influence the processing or target selection of miRNAs [77], adding another level of complexity to deregulation induced by the occurrence of SNPs in miRNAs sequences.

### 4.4. Deregulated Noncoding RNAs Identified at the Expression Level

The search for miRNAs and lncRNAs deregulated in lung cancer has frequently involved expression comparisons between cancer specimens and corresponding normal tissues [78], or between various clinicopathological groupings, such as subtype and therapy response [79–82]. A recent NSCLC study revealed a 41-miRNA signature that could distinguish lung cancer tissues from noncancerous lung tissues, and a 6-miRNA signature that could differentiate the two major NSCLC subtypes: adenocarcinoma (AC) and squamous cell carcinoma (SqCC) [12]. Moreover, miRNA expression studies have identified miRNAs associated with increased metastatic potential [83] and survival [12, 13, 30]. Aberrant ncRNA expression may also be driven by the deregulation of transcription factors. Like *miR-17-92*, expression of the non-coding RNA *H19* has been shown to be induced by MYC in lung carcinomas [84]. Additional lncRNAs whose expression has been shown to be deregulated in lung carcinoma include *H19*, which undergoes loss of imprinting and overexpression [85], *MALAT-1 *[15], cancer up-regulated drug resistant (*CUDR*) [86], and *BC200* [87].

### 4.5. Deregulation of miRNAs by Alterations to Processing Machinery

The general decrease in abundance of mature miRNAs is a common event in cancer and can be at least partially attributed to alterations in the miRNA processing machinery [88]. Described here are such examples that have been documented in lung cancer. Nuclear export of pre-miRNA requires XPO5, which occasionally suffers inactivating mutations resulting in a lower abundance of cytoplasmic miRNA [89]. Inactivating mutations have also been documented in *TARBP2*, encoding TRBP, a cofactor that functions in conjunction with Dicer to cleave pre-miRNA in the cytoplasm [90, 91]. Mutations in these loci appear to be mutually exclusive [91]. In fact, *DICER*, *TARBP2*, and *XPO5* all represent haploinsufficient tumor suppressors in lung cancer [30, 92–94]. Loss of *DICER* has even been linked to shorter time to recurrence and poorly differentiated tumors [30, 95].

## 5. Technologies for the Identification and Quantification of Noncoding RNA

### 5.1. Expression Profiling

Several high-throughput genome wide approaches have been used to discover, establish targets, and predict functions of ncRNAs, including microarrays, serial analysis of gene expression (SAGE), next-generation sequencing, immunoprecipitation (IP)-based, and computational analyses. Tiling path arrays offer some advantages for discovery of ncRNAs over oligonucleotide-based arrays, as they are not dependent on current gene annotations; however, their resolution can be a limiting factor [96]. SAGE libraries, originally used to assess levels of protein-coding transcripts, can be requeried to measure SAGE tag counts that map to lncRNA sequences [97, 98]. Gibb et al. queried SAGE libraries from both cancer and normal tissues, namely, breast, brain, and lung and identified 90 lncRNAs to be specifically deregulated in lung cancers compared to normal lung [98]. Next-generation sequencing technology has facilitated the identification of previously undescribed miRNAs and other small RNAs often missed by the limited depth of traditional sequencing methods [99, 100]. For example, the study by Meiri et al. identified seven novel lung tumor-specific miRNAs, one of which was the star strand of *miR-663* [99]. In addition to identification of novel miRNAs, deep sequencing has also been proven to be a powerful tool for quantifying miRNA expression and defining variation within miRNA sequences. Small RNA sequencing offers a number of significant advantages over microarray platforms. Sequencing approaches are not limited by an *a priori* knowledge of the miRNAs being queried and are not constrained by lower (or upper) limits of detection. The ability to determine absolute expression values that can be compared within and across experiments represents another distinct advantage of sequencing data over the relative quantifications given by microarray measurements [96, 101]. Furthermore, sequencing approaches do not suffer from cross-hybridization artefacts observed in microarray experiments. In order to validate suspected target transcripts and identify enrichment of mRNA subsets potentially regulated by miRNAs, microarray expression analyses and/or sequencing methods are often complemented with IP-based approaches [96].

### 5.2. Computational Prediction

Several computational approaches, based on the conformational characteristics of ncRNAs, have been designed in an attempt to predict non-coding transcripts and their targets [96, 102, 103]. Secondary structure is one of the hallmarks for ncRNA prediction and/or identification software, and most algorithms are based on hairpin structures of precursor forms of miRNAs [104–106]. Different approaches have been developed in order to face the main challenges related with ncRNA research, including secondary structure prediction, comparison, and identification [107]. Computational methods were first focused on folding and prediction of RNA secondary structures, while assuming an RNA molecule is folded using minimum free energy. Some algorithms developed under this context are shown in Table 4. Secondary structure-based approaches can overpass some classes of ncRNAs. For example, lncRNAs can contain structural regions; however, they in general are not densely structured [108]. In this context, accuracy has been improved by comparative analysis based on structure preserving changes of base pairs. Among other methods, the search for ncRNAs can be carried out mainly through sequence and structure similarity, sequence-based alignments, and local searches [108]. Examples of tools using these approaches are also shown in Table 4.

Other computational methods for the identification of ncRNAs and interacting RNA molecules are based on the identification of short conserved motifs in the 3′ UTRs of protein-coding genes, as potential target sequences. Following this, queries are conducted searching for conserved sequences complementary to these motifs [109–111]. Programs based on these characteristics, such as “Pictar,” “TargetScan,” and “MiRanda,” have been used to predict miRNA functions and mRNA targets, resulting in the creation of a number of ncRNA databases [4, 70, 112–114].

### 5.3. Measuring Expression in Archival Tumor Materials

Formalin-fixed, paraffin-embedded (FFPE) samples are the most commonly available clinical specimens for histological and pathological analysis and represent a vast resource of samples for the identification of novel molecular markers as well as therapeutic targets [115, 116]. FFPE tissues have long been considered a challenge for nucleic acid analyses, specifically gene expression studies, as they contain cross-linked and fragmented nucleic acids, and RNA species are often degraded [117]. However, the small size of miRNAs significantly reduces degradation by fixation, and numerous studies have shown miRNAs to be unaffected and well preserved in FFPE samples [118, 119]. Studies have indicated no significant difference in miRNA expression between matched FFPE and fresh frozen (FF) samples, suggesting that FFPE specimens are suitable for miRNA expression analyses [120–123]. Current methods for investigation of miRNA expression levels derived from FFPE specimens include quantitative real-time PCR [70, 116, 124], as well as microarray platforms by Agilent, Affymetrix, and Exiqon [116, 120, 121], all of which have shown comparable results between FFPE and FF tissue.

For lncRNAs, the use of FFPE tissue is not well documented. Due to their longer length, degradation and fragmentation associated with fixation may be an issue, much the same as for mRNA [117]. In an attempt to address these issues, Beck et al. developed a novel method of gene expression profiling termed 3′-end sequencing for expression quantification (3SEQ), which is applied to next-generation sequencing technologies [125]. Comparative analysis of a panel of FFPE and FF desmoid type fibromatosis and solitary fibrous tumor samples demonstrated that 3SEQ of FFPE samples outperformed microarray technologies and was comparable to 3SEQ results from corresponding FF samples. Although further work is still required, 3SEQ may be a useful method for the detection of lncRNAs in FFPE specimens.

## 6. Clinical Application of Non-Coding RNAs

Advancements in our understanding of the mechanisms driving aberrant ncRNA expression in lung cancer and other cancer types may yield significant clinical utility. Commonly overexpressed miRNAs and SNPs within miRNA sequences or target sequences could be exploited diagnostically, as biomarkers of disease. Therapeutically, ncRNAs silenced by methylation could potentially be restored with existing demethylating agents. MiRNAs are well preserved in FFPE tissues and circulate in bodily fluids with substantial stability. They can be used to accurately identify primary and metastatic cancer tissue origins, distinguish lung cancer subtypes, and predict outcome [126]. These qualities highlight their potential as both diagnostic and prognostic biomarkers in lung cancer. Studies examining miRNA levels in blood serum or plasma of patients with cancer appear promising, and there have already been several reports of miRNAs specific to the sera of lung cancer patients that are not detectable in disease-free individuals [127]. Typically, longer RNA species are not stable in blood; however, the short length of miRNAs makes them resistant to degradation and thus robust candidates for blood-based biomarkers [128].

### 6.1. Noncoding RNAs as Diagnostic Biomarkers

Several studies searching for miRNAs capable of separating individuals with lung cancer from those that are lung cancer-free have been conducted. Yu et al. identified a four-miRNA signature (*miR-21, miR-486, miR-375, and miR-200b*) in sputum capable of distinguishing patients with lung adenocarcinoma from normal subjects with reportedly 80.6 sensitivity and 91.7% specificity [129]. Similarly, a five-miRNA signature (*miR-210*, *miR-182*, *miR-486-5p*, *miR-30a*, and *miR-140-3p*) was developed to distinguish squamous cell carcinomas from matched normals [130]. Efforts to improve early detection technologies are ongoing and frequently involve blood-based analyses of miRNA levels. Using Solexa sequencing, Chen et al. detected two overexpressed serum miRNAs (*miR-25* and *miR-223*) that could be used as biomarkers for early detection of NSCLC [127], while another more recent study identified a 10-miRNA signature for the same purpose [131]. Foss et al. recently reported that *miR-1254* and *miR-574-5p* were detected in the sera of patients with early-stage NSCLC compared to controls with a sensitivity and specificity of 82% and 77%, respectively [132]. Early detection has improved with the advent of CT technologies; however, the false positive rate is quite high. To address this issue, a recent study by Shen et al. identified that plasma miRNAs capable of distinguishing between lung cancer and benign nodules in CT-detected solitary pulmonary nodules [133].

Similarly, lung cancer subtypes can also be accurately defined by their characteristic mirRNA expression profiles. For example, *miR-205* is a highly specific marker for squamous cell lung carcinoma, capable of distinguishing squamous from nonsquamous NSCLC with high sensitivity and specificity [10]. Subtypes can be further subcategorized by miRNA expression patterns unique to specific genotypes. In lung adenocarcinoma, *miR-155* is upregulated exclusively in tumors without *KRAS* or *EGFR* mutations. *mir-25* and *miR-21* are upregulated in *EGFR* mutation positive tumors, which are typically found in lung cancer never-smoker cases, while *KRAS* mutation positive tumors are associated with *miR-495* up-regulation [29, 134]. To date, no lncRNAs have demonstrated diagnostic potential in lung cancer; however, diagnostic lncRNAs have been identified in other cancer types. The prostate-specific lncRNA *DD3* is a highly specific marker of prostate cancer detectable in urine [135, 136], whereas the hepatocellular carcinoma-specific lncRNA highly up-regulated in liver cancer (*HULC*), is detectable in the blood of patients with liver cancer [137]. Collectively, these findings support the clinical potential of lncRNAs as diagnostic tools.

### 6.2. Non-Coding RNAs as Prognostic Biomarkers

To date, there are far more prognostic ncRNAs than those for diagnostic purposes, and in this paper, we will focus on only a few of the most well-established biomarkers. *miR-21* is overexpressed in a number of human cancers, including lung cancer, where it has been shown to be an independent negative prognostic factor for overall survival as it stimulates growth and invasion through the inhibition of PTEN [11]. Recently, Saito et al. showed that increased *miR-21* expression is associated with disease progression and survival in stage 1 lung cancer [138]. The Myc-activated miRNA cluster, *miR-17-92*, first identified as potential oncogenes in B-cell lymphoma, plays an important role in lung development, with high expression in embryonic development that declines throughout development into adulthood. Overexpression of this cluster is associated with the inactivation of *RB* and improved tumor vasculature through the inhibition of antiangiogenic thrombosopndin-1 [139, 140]. Hu et al. derived a four miRNA signature that was significantly associated with overall survival of NSCLC patients; this signature derived from serum samples is a demonstration of miRNA stability in blood as well as their potential use as noninvasive biomarkers [141]. Additionallly, miRNA signatures of recurrence free survival in stage I NSCLC patients were established for both NSCLC and AC patients, encompassing 34 and 27 miRNAs, respectively [142]. The scope of lncRNAs as prognostic markers is limited, but growing. *MALAT1* is currently the only lncRNA with prognostic significance in lung cancer and is associated with high metastatic potential and poor patient prognosis in NSCLC [15].

### 6.3. Therapeutic Potential of ncRNAs

miRNAs are implicated in almost every process of lung tumorigenesis, from tumor development to metastasis and drug resistance, underscoring their therapeutic potential. Expression of *let-7* inhibits growth of lung cancer cell lines and xenografts, reducing tumor burden, while lipid-based delivery systems of *miR-34* have been shown to block tumor growth in murine models and downregulate the inhibitor of apoptosis protein, survivin, expression in lung metastases [143, 144]. More recently, *miR-145* was found to inhibit cell proliferation through down-regulation of c-Myc in *EGFR* positive tumors [83], and *miR-200c* abrogated the capacity of metastatic lung adenocarcinoma to undergo epithelial to mesenchymal transition, invade and metastasize. This suggests that ectopic expression of *miR-145 and -200* may be useful as novel therapeutic agents in lung cancer [145]. Expression of *miR-29* displays an antiinvasive and anti-proliferative effect on lung cancer cells in vitro through the restoration of normal patterns of DNA methylation, supporting the notion that miRNAs may have an application as novel demethylating agents [60, 146].

miRNA expression is also known to play a significant role in drug sensitivity and resistance. Sensitivity to cisplatin has been linked with up-regulation of *miR-181a*, whereas resistance is conferred through the up-regulation of *miR-630 *[147]. Improved response and survival following gefitinib treatment has been correlated with loss of *miR-128b *[82], while overexpression of *miR-137, -134, *and* let-7a* has been shown to increase drug sensitivity for a number of anticancer drugs [148]. The clinical utility of lncRNAs as therapeutics has yet to be fully realized; however, overexpression of *CUDR* is associated with resistance to doxorubicin and apoptosis in SqCC cell lines A431 and A10A [86], indicating these transcripts may be useful clinically.

A number of current strategies to manipulate miRNA expression are currently being investigated and tested. These include but are not limited to antagomirs, miRNA sponges and small molecules to reduce miRNA expression, locked nucleic acids (LNAs), lipid-formulated mimics, and adenovirus vectors to over/reexpress down-regulated miRNAs [149, 150]. A number of hurdles remain before miRNAs can be widely established as therapeutic targets. For instance, the targeted delivery of RNA therapeutics to the site of interest, such as primary tumors or their metastases, is a major challenge. Many of the current strategies rely on the increased half-life and stability that chemical modifications offer molecules such as antagomirs and LNAs for systemic circulation and eventual uptake by the target tissue [151–153]. However, this strategy is associated with the possibility of negatively impacting healthy tissues. The ability to specifically deliver RNA therapeutics to the sites of interest would avoid this problem and has been demonstrated in a mouse model of lung metastatic melanoma [44]. Chi et al. developed a liposome-polycation-hyaluronic acid nanoparticle modified with a tumor targeting antibody to deliver the contained siRNA and miRNAs to the lung metastases, resulting in reduced tumor burden. In addition to the challenges of targeted delivery, the possibility of off-target effects is an equally relevant issue and stems from the ability of a single miRNA to target multiple mRNAs. This issue is not simplified by the use of target prediction algorithms, which are far from perfect. While the use of multiple target prediction algorithms improves sensitivity, false positives remain a significant problem, requiring filtering and experimental validation. New methods such as high-throughput sequencing of RNAs isolated by crosslinking immunoprecipitation (HITS-CLIP) are emerging to identify bonafide RISC-associated miRNA-mRNA interactions [154]. Despite these many complicating factors, miRNAs have begun to enter the clinical setting. Preclinical trials are currently underway examining the effectiveness of *let-7* reintroduction into murine NSCLC models, while *miR-122* antagonistic technologies are in Phase II clinical trials for the treatment of Hepatitis C [155].

## 7. Impact of ncRNA Deregulation on Biological Networks

**Figure 1:**
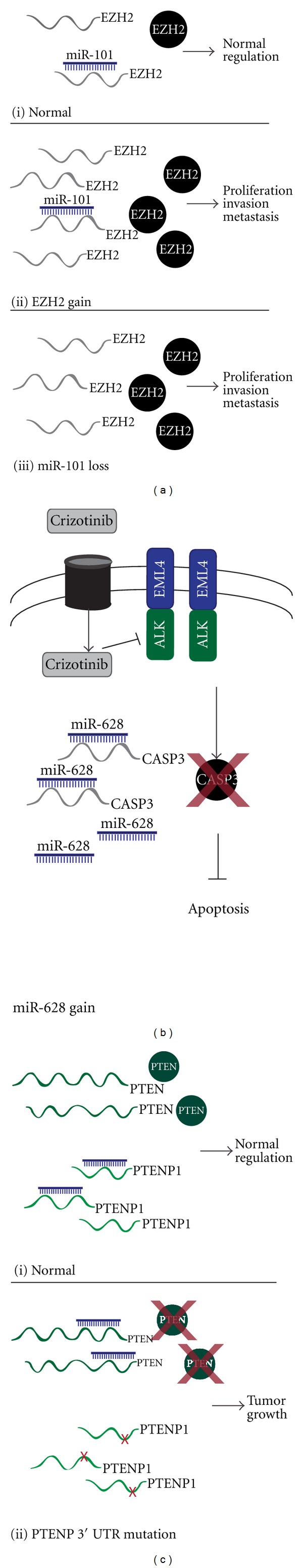
Schematic depiction of ncRNA deregulation and its impact on regulatory proteins. (a) (i) normal levels of EZH2 are maintained by a balance of *EZH2* transcription and *miR-101* regulation. (ii) EZH2 is overexpressed as a result of copy number gain of the *EZH2* locus. (iii) EZH2 is overexpressed as a result of *miR-101* loss. (b) hypothetical scenario of crizotinib resistance. *miR-628* is overexpressed resulting in increased degradation of putative target transcript, *CASP3*, required for crizotinib-induced cell death. (c) (i) normal levels of PTEN are maintained through the ability of its pseudogene, *PTENP1*, to act as a miRNA sponge. (ii) mutation in the 3′-UTR of *PTENP1* results in loss of miRNA binding and redirection of the miRNA to degrade *PTEN*.

Significant advances in our understanding of lung cancer biology will result from an improved understanding of the interplay between the deregulation of protein-coding genes and ncRNAs. Such an example is well illustrated by the relationship between *miR-101* and *EZH2* (Figure 1(a)). *miR-101* is frequently deleted, with a higher preponderance of loss in NSCLC as compared to SCLC [156], while EZH2, a subunit of the PRC2 complex, often experiences gain, overexpression, and activating mutations. *EZH2* gain is also considered a negative prognostic factor in lung cancer as it promotes proliferation, invasion, and metastasis through the transcriptional repression of target genes such as *CDKN2A*. *EZH2* is a target of *miR-101 *[157], with loss of *miR-101* resulting in derepression of *EZH2 *expression, and providing yet another mechanism of EZH2 activation. Importantly, this relationship informs us of mechanisms of oncogene activation beyond copy number gain, mutation, and hypomethylation. This provides evidence that identification of novel cancer-related genes may result from examination of ncRNA deregulation, which may have been missed by studying conventional means of disruption.

As miRNAs are major regulators of gene signalling pathways, it is not surprising that they have been shown to be deregulated in association with specific drug response phenotypes. A multidimensional analysis of a panel of lung cancer cell lines examining miRNA copy number, expression and mRNA expression found gain and overexpression of *miR-628* to be associated with crizotinib drug resistance [80] (Figure 1(b)). Crizotinib-induced cell death occurs through activation of the caspase-3 pathway, and interestingly, one of the predicted targets of *miR-628* was *CASP3* [158]. These data suggest that *miR-628* may play a critical role in crizotinib resistance through the repression of a key effector molecule required for drug function. The addition of miRNAs, and eventually lncRNAs, to gene networks will further our understanding of the biological mechanisms governing drug response, and potentially influence treatment choices or even identify novel therapeutic targets.

These two examples represent the classical view of miRNA repression of protein-coding transcripts. However, a growing area of research pertains to cellular interaction networks, namely, the interaction between miRNAs and lncRNAs. LncRNAs can act as natural “miRNA sponges” reducing levels of free miRNAs and relieving inhibition of other target transcripts [159]. One such example is *HULC*, an lncRNA that contains a cAMP response element binding protein (CREB) binding site in its promoter. *HULC* creates an autoregulatory loop by acting as a molecular decoy that sequesters *miR-372 *and prevents repression of PRKACB, a kinase that targets CREB. Through this mechanism *HULC* facilitates CREB induction, which subsequently leads to increased *HULC* transcription [160]. Another example of lncRNA-miRNA regulation was identified in prostate cancer. The 3′ UTR of the tumor suppressor *PTEN*, frequently lost in prostate and other cancer types, shares sequence similarity with the lncRNA *PTENP1*. The homology in 3′ UTR sequences results in common targeting by the same miRNAs, thus *PTENP1* can act as a molecular sponge for miRNAs that target *PTEN, *limiting *PTEN* repression by miRNAs (Figure 1(c)). Mutations to the *PTENP1* 3′ UTR disrupt miRNA binding eliminating the protective ability of the transcript and leading to repression of *PTEN* and promotion of tumor growth [161, 162]. This is a novel finding, with no similar examples described in lung cancer. Further study into miRNA-lncRNA relationships could lead to the identification of novel miRNA, lncRNA, and protein-coding gene signalling triads.

## 8. Conclusions

ncRNAs play a role in nearly every biological process and therefore have the potential to serve as diagnostic and prognostic biomarkers as well as therapeutic targets. Findings from recent studies strongly support this notion, with ncRNAs being implicated in survival, prognosis, and drug response while also being capable of discerning cancerous from benign lesions and discriminating between subtypes of lung cancer. It is evident that a comprehensive understanding of tumor biology must therefore include both coding and non-coding transcripts. Only through the inclusion of these transcripts in molecular studies it will be possible to better understand tumor biology and human disease.

## Figures and Tables

**Table 1:** Classes of human non-coding RNAs.

Type	Class	Characteristics and function	References
Small ncRNA (<200 nt)	Small Interfering RNAs (siRNAs)	21-22 nt double-stranded RNAs produced by Dicer and involved in gene silencing and viral defence	[163]
	microRNAs (miRNAs)	18–25 nt RNAs that modulate gene expression posttranscriptionally	[163, 164]
	Transfer RNAs (tRNA)	An adaptor molecule with an inverted L structure involved in translation of mRNA into protein	[165]
	PIWI-interacting RNAs (piRNAs)	Dicer independent 26–31 nt RNAs located in the germline and adjacent somatic cells, involved in germline development and stability through the regulation of transposons	[163]
	Small nucleolar RNAs (snoRNAs)	Guide molecules for modification and processing of rRNA, specifically site-specific methylation and pseudouridylation	[164]
	microRNA-offset RNAs (moRNAs)	RNAs derived from the ends of pre-miRNAs, predominantly from the 5′ end, independent of the mature miRNA. The function of moRNAs are currently unknown	[16]
	Ribosomal 5.8S	Transcribed by pol I as a part of the 45S precursor, 5.8S is a component of the large ribosomal subunit in eukaryotes, and thus involved in protein translation	[166]
	Promoter-associated short RNAs (PASRs)	Transcripts within a few hundred bases of protein coding or noncoding transcription start site that may regulate gene expression	[167]

Long ncRNA (>200 nt)	Long ncRNA	A broad class of RNAs > 200 nt with functions in epigenetic regulation, splicing, and cellular localization	[40]
	Transcribed ultraconserved regions (T-UCR)	Non-coding sequences 100% conserved among humans, mice, and rats, with roles in the regulation of alternative splicing and gene expression, and altered in a number of human cancers	[168]
	Pseudogenes	Nonfunctional sequences of genomic DNA originally derived from functional genes but with mutations or premature stop codons that prevent their expression. Known to regulate gene expression and recombination	[161, 169]
	Promoter associated long RNAs (PARs)	Transcripts 250–500 nt long within a few hundred bases of protein coding or non coding transcription start sites that may regulate gene expression	[167]
	Antisense RNAs	Single stranded RNA complementary to a transcribed mRNA, capable of binding and blocking translation of its complementary mRNA, and promoting target decay.	[170]

**Table 2:** Involvement of miRNAs in lung cancer and technologies used for identification.

miRNA affected	Significance	Technology	Source tissue	References
Overexpression of miR-155, miR-21, and miR-106a. Decreased expression of let-7a	Prognostic biomarker of adenocarcinoma patient survival	Oligonucleotide microchip	Primary LC cases and corresponding noncancerous tissues	[12]
Overexpression of miR-21	Candidate for molecular targets in treatment for LC in never-smokers	miRNA microarray assay on a CodeLink platform (miRNA oligo probe)	Matched pairs of LC and noncancerous lung tissues from never-smokers	[134]
Decreased expression of let-7	Shortened postoperative survival in NSCLC	RT-PCR	Tumor specimens	[30]
Decreased expression of miRNA-451	Expression negatively associated with lymph node metastasis, the stage of TNM classification and poor prognosis of NSCLC patients	qPCR, confirmed by northern blot analysis	Fresh tissue of NSCLC samples and the adjacent histologically normal tissue.	[171]
Overexpression of miR-92-1	Regulation of RAB14 gene at the translational level. This might cause a decrease in the lung surfactant secretion, and loss of the protection of lung cells against external carcinogens	2D electrophoresis profiling and mass spectrometric analysis	SBC-3 cell line	[172]
Decreased expression of miR-30a	May function as a tumor suppressor, by targeting Snai1 and inhibiting migration, invasion, and metastasis	qPCR	A549 cell line and fresh snap-frozen surgical specimens of tumor tissues and of the corresponding normal specimens	[173]
Overexpression of miR-21	Overexpressed in tumor tissues relative to adjacent nontumor tissues. Negative regulation of PTEN and promotion of cellular growth and invasion in NSCLC cells	qRT-PCR	Paired NSCLC and adjacent non-tumor lung tissues	[174]
Overexpression of miR-126	Inhibition of tumor growth in vivo by targeting EGFL7	Flow cytometry assay, qRT-PCR, and Western blot	A549 cell line	[175]
Decreased expression of miR-133B	Increased apoptosis in response to gemcitabine and reduced MCL-1 and BCL2L2 expression	Quantitative-reverse transcriptase (q-RT) PCR profiling	Frozen lung tumors (adenocarcinoma) and noninvolved adjacent lung and LC cell lines	[78]
Overexpression and gain of miR*-*17-92	Enhanced cell proliferation	Northern blot confirmed with RT-PCR, followed by southern blot	SCLC tissue and cell lines	[55]
Loss of miR-1	Inhibition of cell proliferation and invasion in vitro, and tumor growth in vivo, by targeting MET and FoxP1	qRT-PCR	Lung cancer tissue and cell lines	[59]
Hypermethylation of miR-34a	Avoidance of senescence	Methylation-specific PCR followed by qRT-PCR	Lung cancer cell lines	[57]
Overexpression of miR-25 and miR-223	Biomarker of NSCLC found in sera of NSCLC patients, but not in that of healthy donors	Solexa sequencing	Sera from NSCLC compared to healthy donor controls	[127]
Overexpression of miR-21 and miR-210, decreased expression of miR-486-5p	Biomarkers of malignant nodules identified by CT	qRT-PCR	Plasma from patients with malignant nodules compared to those with benign nodules and healthy controls	[133]

**Table 3:** Involvement of lncRNAs in lung cancer and technologies used for identification.

lncRNA	Significance	Technology	Source tissue	Reference
Overexpression of MALAT1	Predict metastasis and survival in early-stage NSCLC	Subtractive hybridization method, sequencing and quantitative RT-PCR	Shock frozen primary nonsmall cell lung tumors	[15]
Deregulated expression of BC200	Detectable at significant levels in tumors. Normal tissue from the same patient was found to be BC200-negative	In situ hybridization	Tumour and normal tissue frozen in liquid nitrogen	[87]
Overexpression of H19	Loss of imprinting in lung adenocarcinoma	RT-PCR	LC tissue and normal lung	[176]

**Table 4:** Examples of computational approaches used for ncRNA characterization.

Method	Brief description	Reference
Secondary structure		
MFOLD	Folding prediction using a thermodynamic model, returning a structure of minimal free energy (MFE)	[177]
RNAfold		[103]
PKNOTS	Algorithm which finds optimal pseudoknotted RNA structures	[178]
pknotsRG	Finds the best RNA structure including the pseudoknot (based on MFE-model)	[179, 180]

Sequence similarity search		
INFERNAL	Generates consensus RNA secondary structure, then searches for homologous RNAs, or creates new sequence- and structure-based multiple sequence alignments.	[181]

Sequence-based alignments		
RNAz	*Performs de novo *searches for RNA structure	[182]
qRNA	Predicts structured RNAs from sequence alignments (only works on pair-wise alignments)	[183]
Evofold	Functional RNA-structure identification in multiple sequence alignments	[184]
Dynalign	A free energy minimization algorithm for joint alignment and secondary structure prediction	[185]

Local searches		
FOLDALIGN	Alignment of RNA sequences and selection of subsets containing the most significant alignments.	[186]
CMfinder	Finds conserved RNA motifs in a set of unaligned sequences	[187]

## References

[1] Ponting CP, Belgard TG (2010). Transcribed dark matter: meaning or myth?. *Human Molecular Genetics*.

[2] Birney E, Stamatoyannopoulos JA, Dutta A (2007). Identification and analysis of functional elements in 1% of the human genome by the ENCODE pilot project. *Nature*.

[3] Carninci P, Kasukawa T, Katayama S (2005). The transcriptional landscape of the mammalian genome. *Science*.

[4] Gibb EA, Brown CJ, Lam WL (2011). The functional role of long non-coding RNA in human carcinomas. *Molecular Cancer*.

[5] Hung T, Chang HY (2010). Long noncoding RNA in genome regulation: prospects and mechanisms. *RNA Biology*.

[6] Taft RJ, Pang KC, Mercer TR, Dinger M, Mattick JS (2010). Non-coding RNAs: regulators of disease. *Journal of Pathology*.

[7] Jemal A, Siegel R, Ward E, Hao Y, Xu J, Thun MJ (2009). Cancer statistics, 2009. *CA Cancer Journal for Clinicians*.

[8] Jemal A, Siegel R, Xu J, Ward E (2010). Cancer statistics, 2010. *CA Cancer Journal for Clinicians*.

[9] Sato M, Shames DS, Gazdar AF, Minna JD (2007). A translational view of the molecular pathogenesis of lung cancer. *Journal of Thoracic Oncology*.

[10] Aharonov R, Lebanony D, Benjamin H (2009). Diagnostic assay based on hsa-miR-205 expression distinguishes squamous from nonsquamous non-small-cell lung carcinoma. *Journal of Clinical Oncology*.

[11] Markou A, Tsaroucha EG, Kaklamanis L, Fotinou M, Georgoulias V, Lianidou ES (2008). Prognostic value of mature MicroRNA-21 and MicroRNA-205 overexpression in non-small cell lung cancer by quantitative real-time RT-PCR. *Clinical Chemistry*.

[12] Yanaihara N, Caplen N, Bowman E (2006). Unique microRNA molecular profiles in lung cancer diagnosis and prognosis. *Cancer Cell*.

[13] Yu SL, Chen HY, Chang GC (2008). MicroRNA signature predicts survival and relapse in lung cancer. *Cancer Cell*.

[14] Calin GA, Croce CM (2006). MicroRNA signatures in human cancers. *Nature Reviews Cancer*.

[15] Ji P, Diederichs S, Wang W (2003). MALAT-1, a novel noncoding RNA, and thymosin *β*4 predict metastasis and survival in early-stage non-small cell lung cancer. *Oncogene*.

[16] Bortoluzzi S, Biasiolo M, Bisognin A (2011). microRNA-offset RNAs (moRNAs): by-product spectators or functional players?. *Trends in Molecular Medicine*.

[17] Cheng J, Guo JM, Xiao BX (2011). PiRNA, the new non-coding RNA, is aberrantly expressed in human cancer cells. *Clinica Chimica Acta*.

[18] Bartel DP (2004). MicroRNAs: genomics, biogenesis, mechanism, and function. *Cell*.

[19] Lytle JR, Yario TA, Steitz JA (2007). Target mRNAs are repressed as efficiently by microRNA-binding sites in the 5′ UTR as in the 3′ UTR. *Proceedings of the National Academy of Sciences of the United States of America*.

[20] Winter J, Diederichs S (2011). MicroRNA biogenesis and cancer. *Methods in Molecular Biology*.

[21] Grimson A, Farh KKH, Johnston WK, Garrett-Engele P, Lim LP, Bartel DP (2007). MicroRNA targeting specificity in mammals: determinants beyond seed pairing. *Molecular Cell*.

[22] Yekta S, Shih IH, Bartel DP (2004). MicroRNA-directed cleavage of HOXB8 mRNA. *Science*.

[23] Shyu AB, Wilkinson MF, Van Hoof A (2008). Messenger RNA regulation: to translate or to degrade. *The EMBO Journal*.

[24] Iorio MV, Croce CM (2009). MicroRNAs in cancer: small molecules with a huge impact. *Journal of Clinical Oncology*.

[25] Eulalio A, Rehwinkel J, Stricker M (2007). Target-specific requirements for enhancers of decapping in miRNA-mediated gene silencing. *Genes & Development*.

[26] Kozomara A, Griffiths-Jones S (2011). MiRBase: integrating microRNA annotation and deep-sequencing data. *Nucleic Acids Research*.

[27] Zhang L, Huang J, Yang N (2006). microRNAs exhibit high frequency genomic alterations in human cancer. *Proceedings of the National Academy of Sciences of the United States of America*.

[28] Calin GA, Sevignani C, Dumitru CD (2004). Human microRNA genes are frequently located at fragile sites and genomic regions involved in cancers. *Proceedings of the National Academy of Sciences of the United States of America*.

[29] Dacic S, Kelly L, Shuai Y, Nikiforova MN (2010). MiRNA expression profiling of lung adenocarcinomas: correlation with mutational status. *Modern Pathology*.

[30] Takamizawa J, Konishi H, Yanagisawa K (2004). Reduced expression of the *let*-7 microRNAs in human lung cancers in association with shortened postoperative survival. *Cancer Research*.

[31] Garofalo M, Di Leva G, Romano G (2009). *miR-221&222* regulate TRAIL resistance and enhance tumorigenicity through PTEN and TIMP3 downregulation. *Cancer Cell*.

[32] Gallardo E, Navarro A, Viñolas N (2009). miR-34a as a prognostic marker of relapse in surgically resected non-small-cell lung cancer. *Carcinogenesis*.

[33] Jiang L, Huang Q, Zhang S (2010). Hsa-miR-125a-3p and hsa-miR-125a-5p are downregulated in non-small cell lung cancer and have inverse effects on invasion and migration of lung cancer cells. *BMC Cancer*.

[34] Wang X, Ling C, Bai Y, Zhao J (2011). MicroRNA-206 is associated with invasion and metastasis of lung cancer. *Anatomical Record*.

[35] Lin PY, Yu SL, Yang PC (2010). MicroRNA in lung cancer. *British Journal of Cancer*.

[36] Wapinski O, Chang HY (2011). Long noncoding RNAs and human disease. *Trends in Cell Biology*.

[37] Gong C, Maquat LE (2011). LncRNAs transactivate STAU1-mediated mRNA decay by duplexing with 39 UTRs via Alu eleme. *Nature*.

[38] Kotake Y, Nakagawa T, Kitagawa K (2011). Long non-coding RNA ANRIL is required for the PRC2 recruitment to and silencing of p15 INK4B tumor suppressor gene. *Oncogene*.

[39] Gupta RA, Shah N, Wang KC (2010). Long non-coding RNA HOTAIR reprograms chromatin state to promote cancer metastasis. *Nature*.

[40] Mercer TR, Dinger ME, Mattick JS (2009). Long non-coding RNAs: insights into functions. *Nature Reviews Genetics*.

[41] Mercer TR, Dinger ME, Sunkin SM, Mehler MF, Mattick JS (2008). Specific expression of long noncoding RNAs in the mouse brain. *Proceedings of the National Academy of Sciences of the United States of America*.

[42] Cabili MN, Trapnell C, Goff L (2011). Integrative annotation of human large intergenic noncoding RNAs reveals global properties and specific subclasses. *Genes & Development*.

[43] Guttman M, Donaghey J, Carey BW (2011). lincRNAs act in the circuitry controlling pluripotency and differentiation. *Nature*.

[44] Castle JC, Armour CD, Löwer M (2010). Digital genome-wide ncRNA expression, including SnoRNAs, across 11 human tissues using polya-neutral amplification. *PLoS One*.

[45] Lipovich L, Johnson R, Lin C-Y (2010). MacroRNA underdogs in a microRNA world: evolutionary, regulatory, and biomedical significance of mammalian long non-protein-coding RNA. *Biochimica et Biophysica Acta*.

[46] Beysen D, Raes J, Leroy BP (2005). Deletions involving long-range conserved nongenic sequences upstream and downstream of FOXL2 as a novel disease-causing mechanism in blepharophimosis syndrome. *American Journal of Human Genetics*.

[47] Lukiw WJ, Handley P, Wong L, McLachlan DRC (1992). BC200 RNA in normal human neocortex, non-Alzheimer dementia (NAD), and senile dementia of the Alzheimer type (AD). *Neurochemical Research*.

[48] Jin P, Warren ST (2000). Understanding the molecular basis of fragile X syndrome. *Human Molecular Genetics*.

[49] Rinn JL, Kertesz M, Wang JK (2007). Functional demarcation of active and silent chromatin domains in human HOX loci by noncoding RNAs. *Cell*.

[50] Tripathi V, Ellis JD, Shen Z (2010). The nuclear-retained noncoding RNA MALAT1 regulates alternative splicing by modulating SR splicing factor phosphorylation. *Molecular Cell*.

[51] Osada H, Takahashi T (2011). *let*-7 and miR-17-92: small-sized major players in lung cancer development. *Cancer Science*.

[52] Schultz J, Lorenz P, Gross G, Ibrahim S, Kunz M (2008). MicroRNA *let*-7b targets important cell cycle molecules in malignant melanoma cells and interferes with anchorage-independent growth. *Cell Research*.

[53] Esquela-Kerscher A, Trang P, Wiggins JF (2008). The *let*-7 microRNA reduces tumor growth in mouse models of lung cancer. *Cell Cycle*.

[54] Johnson CD, Esquela-Kerscher A, Stefani G (2007). The *let*-7 microRNA represses cell proliferation pathways in human cells. *Cancer Research*.

[55] Hayashita Y, Osada H, Tatematsu Y (2005). A polycistronic MicroRNA cluster, miR-17-92, is overexpressed in human lung cancers and enhances cell proliferation. *Cancer Research*.

[56] Diederichs S, Haber DA (2006). Sequence variations of microRNAs in human cancer: alterations in predicted secondary structure do not affect processing. *Cancer Research*.

[57] Lodygin D, Tarasov V, Epanchintsev A (2008). Inactivation of miR-34a by aberrant CpG methylation in multiple types of cancer. *Cell Cycle*.

[58] Brueckner B, Stresemann C, Kuner R (2007). The human *let*-7a-3 locus contains an epigenetically regulated microRNA gene with oncogenic function. *Cancer Research*.

[59] Nasser MW, Datta J, Nuovo G (2008). Down-regulation of micro-RNA-1 (miR-1) in lung cancer: suppression of tumorigenic property of lung cancer cells and their sensitization to doxorubicin-induced apoptosis by miR-1. *The Journal of Biological Chemistry*.

[60] Fabbri M, Garzon R, Cimmino A (2007). MicroRNA-29 family reverts aberrant methylation in lung cancer by targeting DNA methyltransferases 3A and 3B. *Proceedings of the National Academy of Sciences of the United States of America*.

[61] Saunders MA, Liang H, Li WH (2007). Human polymorphism at microRNAs and microRNA target sites. *Proceedings of the National Academy of Sciences of the United States of America*.

[62] Pasquinelli AE, Reinhart BJ, Slack F (2000). Conservation of the sequence and temporal expression of *let*-7 heterochronic regulatory RNA. *Nature*.

[63] Wu M, Jolicoeur N, Li Z (2008). Genetic variations of microRNAs in human cancer and their effects on the expression of miRNAs. *Carcinogenesis*.

[64] Hu Z, Chen J, Tian T (2008). Genetic variants of miRNA sequences and non-small cell lung cancer survival. *The Journal of Clinical Investigation*.

[65] Tian T, Shu Y, Chen J (2009). A functional genetic variant in microRNA-196a2 is associated with increased susceptibility of lung cancer in Chinese. *Cancer Epidemiology Biomarkers and Prevention*.

[66] Hu Z, Shu Y, Chen Y (2011). Genetic polymorphisms in the precursor microRNA flanking region and non-small cell lung cancer survival. *American Journal of Respiratory and Critical Care Medicine*.

[67] Kim MJ, Yoo SS, Choi YY, Park JY (2010). A functional polymorphism in the pre-microRNA-196a2 and the risk of lung cancer in a Korean population. *Lung Cancer*.

[68] Mishra PJ, Mishra PJ, Banerjee D, Bertino JR (2008). MiRSNPs or MiR-polymorphisms, new players in microRNA mediated regulation of the cell: introducing microRNA pharmacogenomics. *Cell Cycle*.

[69] Wang F, Ma YL, Zhang P (2011). A genetic variant in microRNA-196a2 is associated with increased cancer risk: a meta-analysis. *Molecular Biology Reports*.

[70] Betel D, Wilson M, Gabow A, Marks DS, Sander C (2008). The microRNA.org resource: targets and expression. *Nucleic Acids Research*.

[71] Campayo M, Navarro A, Viñolas N (2011). A dual role for KRT81: a miR-SNP associated with recurrence in Non-Small-Cell lung cancer and a novel marker of squamous cell lung carcinoma. *PLoS One*.

[72] Rotunno M, Zhao Y, Bergen AW (2010). Inherited polymorphisms in the RNA-mediated interference machinery affect microRNA expression and lung cancer survival. *British Journal of Cancer*.

[73] Fang X, Wu C, Chang J (2011). Genetic variation in an miRNA-1827 binding site in MYCL1 alters susceptibility to small-cell lung cancer. *Cancer Research*.

[74] Chin LJ, Ratner E, Leng S (2008). A SNP in a *let*-7 microRNA complementary site in the KRAS 3′ untranslated region increases non-small cell lung cancer risk. *Cancer Research*.

[75] Hong YS (2011). Association between microRNA196a2 rs11614913 genotypes and the risk of non-small cell lung cancer in Korean population. *Journal of Preventive Medicine and Public Health*.

[76] Chu H, Wang M, Shi D (2011). Hsa-miR-196a2 Rs11614913 polymorphism contributes to cancer susceptibility: evidence from 15 case-control studies. *PLoS One*.

[77] Harfe BD (2005). MicroRNAs in vertebrate development. *Current Opinion in Genetics and Development*.

[78] Crawford M, Batte K, Yu L (2009). MicroRNA 133B targets pro-survival molecules MCL-1 and BCL2L2 in lung cancer. *Biochemical and Biophysical Research Communications*.

[79] Du L, Schageman JJ, Irnov (2010). MicroRNA expression distinguishes SCLC from NSCLC lung tumor cells and suggests a possible pathological relationship between SCLCs and NSCLCs. *Journal of Experimental and Clinical Cancer Research*.

[80] Enfield KSS, Stewart GL, Pikor LA (2011). MicroRNA gene dosage alterations and drug response in lung cancer. *Journal of Biomedicine and Biotechnology*.

[81] Hummel R, Hussey DJ, Haier J (2010). MicroRNAs: predictors and modifiers of chemo- and radiotherapy in different tumour types. *European Journal of Cancer*.

[82] Weiss GJ, Bemis LT, Nakajima E (2008). EGFR regulation by microRNA in lung cancer: correlation with clinical response and survival to gefitinib and EGFR expression in cell lines. *Annals of Oncology*.

[83] Gibbons DL, Lin W, Creighton CJ (2009). Contextual extracellular cues promote tumor cell EMT and metastasis by regulating miR-200 family expression. *Genes & Development*.

[84] Barsyte-Lovejoy D, Lau SK, Boutros PC (2006). The c-Myc oncogene directly induces the H19 noncoding RNA by allele-specific binding to potentiate tumorigenesis. *Cancer Research*.

[85] Kondo M, Takahashi T (1996). Altered genomic imprinting in the IGF2 and H19 genes in human lung cancer. *Nippon Rinsho*.

[86] Wing PT, Wong TWL, Cheung AHH, Co CNN, Tim TK (2007). Induction of drug resistance and transformation in human cancer cells by the noncoding RNA CUDR. *RNA*.

[87] Chen W, Böcker W, Brosius J, Tiedge H (1997). Expression of neural BC200 RNA in human tumours. *Journal of Pathology*.

[88] Kumar MS, Lu J, Mercer KL, Golub TR, Jacks T (2007). Impaired microRNA processing enhances cellular transformation and tumorigenesis. *Nature Genetics*.

[89] Melo SA, Moutinho C, Ropero S (2010). A genetic defect in exportin-5 traps precursor MicroRNAs in the nucleus of cancer cells. *Cancer Cell*.

[90] Chendrimada TP, Gregory RI, Kumaraswamy E (2005). TRBP recruits the Dicer complex to Ago2 for microRNA processing and gene silencing. *Nature*.

[91] Großhans H, Büssing I (2010). MicroRNA biogenesis takes another single hit from microsatellite instability. *Cancer Cell*.

[92] Melo SA, Ropero S, Moutinho C (2009). A TARBP2 mutation in human cancer impairs microRNA processing and DICER1 function. *Nature Genetics*.

[93] Kumar MS, Pester RE, Chen CY (2009). Dicer1 functions as a haploinsufficient tumor suppressor. *Genes & Development*.

[94] Lambertz I, Nittner D, Mestdagh P (2010). Monoallelic but not biallelic loss of Dicer1 promotes tumorigenesis in vivo. *Cell Death and Differentiation*.

[95] Karube Y, Tanaka H, Osada H (2005). Reduced expression of Dicer associated with poor prognosis in lung cancer patients. *Cancer Science*.

[96] Wilbert ML, Yeo GW (2011). Genome-wide approaches in the study of microRNA biology. *Wiley Interdisciplinary Reviews*.

[97] Gibb EA, Enfield KSS, Stewart GL (2011). Long non-coding RNAs are expressed in oral mucosa and altered in oral premalignant lesions. *Oral Oncology*.

[98] Gibb EA, Vucic EA, Enfield KSS (2011). Human cancer long non-coding RNA transcriptomes. *PLoS One*.

[99] Meiri E, Levy A, Benjamin H (2010). Discovery of microRNAs and other small RNAs in solid tumors. *Nucleic Acids Research*.

[100] Lu C, Tej SS, Luo S, Haudenschild CD, Meyers BC, Green PJ (2005). Genetics: elucidation of the small RNA component of the transcriptome. *Science*.

[101] Garnis C, Buys TPH, Lam WL (2004). Genetic alteration and gene expression modulation during cancer progression. *Molecular Cancer*.

[102] Lim LP, Lau NC, Weinstein EG (2003). The microRNAs of Caenorhabditis elegans. *Genes & Development*.

[103] Hofacker IL (2003). Vienna RNA secondary structure server. *Nucleic Acids Research*.

[104] Lau NC, Lim LP, Weinstein EG, Bartel DP (2001). An abundant class of tiny RNAs with probable regulatory roles in Caenorhabditis elegans. *Science*.

[105] Lagos-Quintana M, Rauhut R, Lendeckel W, Tuschl T (2001). Identification of novel genes coding for small expressed RNAs. *Science*.

[106] Hutvágner G, McLachlan J, Pasquinelli AE, Bálint É, Tuschl T, Zamore PD (2001). A cellular function for the RNA-interference enzyme dicer in the maturation of the *let* -7 small temporal RNA. *Science*.

[107] MacHado-Lima A, Del Portillo HA, Durham AM (2008). Computational methods in noncoding RNA research. *Journal of Mathematical Biology*.

[108] Gorodkin J, Hofacker IL (2011). From structure prediction to genomic screens for novel non-coding RNAs. *PLoS Computational Biology*.

[109] Chan CS, Elemento O, Tavazoie S (2005). Revealing posttranscriptional regulatory elements through network-level conservation. *PLoS Computational Biology*.

[110] Adai A, Johnson C, Mlotshwa S (2005). Computational prediction of miRNAs in Arabidopsis thaliana. *Genome Research*.

[111] Xie X, Lu J, Kulbokas EJ (2005). Systematic discovery of regulatory motifs in human promoters and 3′ UTRs by comparison of several mammals. *Nature*.

[112] Lewis BP, Burge CB, Bartel DP (2005). Conserved seed pairing, often flanked by adenosines, indicates that thousands of human genes are microRNA targets. *Cell*.

[113] Wang X, El Naqa IM (2008). Prediction of both conserved and nonconserved microRNA targets in animals. *Bioinformatics*.

[114] John B, Enright AJ, Aravin A, Tuschl T, Sander C, Marks DS (2004). Human MicroRNA targets. *PLoS Biology*.

[115] Lehmann U, Kreipe H (2001). Real-time PCR analysis of DNA and RNA extracted from formalin-fixed and paraffin-embedded biopsies. *Methods*.

[116] Leite KRM, Canavez JMS, Reis ST (2011). miRNA analysis of prostate cancer by quantitative real time PCR: comparison between formalin-fixed paraffin embedded and fresh-frozen tissue. *Urologic Oncology*.

[117] Cronin M, Pho M, Dutta D (2004). Evaluation and validation of total RNA extraction methods for microRNA expression analyses in formalin-fixed, paraffin-embedded tissues. *American Journal of Pathology*.

[118] Doleshal M, Magotra AA, Choudhury B, Cannon BD, Labourier E, Szafranska AE (2008). Evaluation and validation of total RNA extraction methods for microRNA expression analyses in formalin-fixed, paraffin-embedded tissues. *Journal of Molecular Diagnostics*.

[119] Klopfleisch R, Weiss AT, Gruber AD (2011). Excavation of a buried treasure—DNA, mRNA, miRNA and protein analysis in formalin fixed, paraffin embedded tissues. *Histology and Histopathology*.

[120] Zhang X, Chen J, Radcliffe T, LeBrun DP, Tron VA, Feilotter H (2008). An array-based analysis of microRNA expression comparing matched frozen and formalin-fixed paraffin-embedded human tissue samples. *Journal of Molecular Diagnostics*.

[121] Xi Y, Nakajima G, Gavin E (2007). Systematic analysis of microRNA expression of RNA extracted from fresh frozen and formalin-fixed paraffin-embedded samples. *RNA*.

[122] Hasemeier B, Christgen M, Kreipe H, Lehmann U (2008). Reliable microRNA profiling in routinely processed formalin-fixed paraffin-embedded breast cancer specimens using fluorescence labelled bead technology. *BMC Biotechnology*.

[123] Siebolts U, Vamholt H, Drebber U, Dienes HP, Wickenhauser C, Odenthal M (2009). Tissues from routine pathology archives are suitable for microRNA analyses by quantitative PCR. *Journal of Clinical Pathology*.

[124] Hui AB, Shi W, Boutros PC (2009). Robust global micro-RNA profiling with formalin-fixed paraffin-embedded breast cancer tissues. *Laboratory Investigation*.

[125] Beck AH, Weng Z, Witten DM (2010). 3′-end Sequencing for Expression Quantification (3SEQ) from archival tumor samples. *PLoS One*.

[126] Hubaux R, Becker-Santos DD, Enfield KSS, Lam S, Lam WL, Martinez VD MicroRNAs as biomarkers for clinical features of lung cancer.

[127] Chen X, Ba Y, Ma L (2008). Characterization of microRNAs in serum: a novel class of biomarkers for diagnosis of cancer and other diseases. *Cell Research*.

[128] Chin LJ, Slack FJ (2008). A truth serum for cancer microRNAs have major potential as cancer biomarkers. *Cell Research*.

[129] Yu L, Todd NW, Xing L (2010). Early detection of lung adenocarcinoma in sputum by a panel of microRNA markers. *International Journal of Cancer*.

[130] Tan X,  Qin W, Zhang L A Five-microRNA signature for squamous cell lung carcinoma (SCC) diagnosis and Hsa-miR-31 for SCC prognosis.

[131] Chen X, Hu Z, Wang W (2011). Identification of ten serum microRNAs from a genome-wide serum microRNA expression profile as novel noninvasive biomarkers for nonsmall cell lung cancer diagnosis. *International Journal of Cancer*.

[132] Foss KM, Sima C, Ugolini D, Neri M, Allen KE, Weiss GJ (2011). MiR-1254 and miR-574-5p: serum-based microRNA biomarkers for early-stage non-small cell lung cancer. *Journal of Thoracic Oncology*.

[133] Shen J, Liu Z, Todd NW (2011). Diagnosis of lung cancer in individuals with solitary pulmonary nodules by plasma microRNA biomarkers. *BMC Cancer*.

[134] Seike M, Goto A, Okano T (2009). MiR-21 is an EGFR-regulated anti-apoptotic factor in lung cancer in never-smokers. *Proceedings of the National Academy of Sciences of the United States of America*.

[135] Tinzl M, Marberger M, Horvath S, Chypre C (2004). DD3PCA3 RNA analysis in urine—a new perspective for detecting prostate cancer. *European Urology*.

[136] Hessels D, Klein Gunnewiek JMT, Van Oort I (2003). DD3PCA3-based molecular urine analysis for the diagnosis of prostate cancer. *European Urology*.

[137] Panzitt K, Tschernatsch MMO, Guelly C (2007). Characterization of HULC, a novel gene with striking up-regulation in hepatocellular carcinoma, as noncoding RNA. *Gastroenterology*.

[138] Saito M, Schetter AJ, Mollerup S (2011). The association of microRNA expression with prognosis and progression in early-stage, non-small cell lung adenocarcinoma: a retrospective analysis of three cohorts. *Clinical Cancer Research*.

[139] Ebi H, Sato T, Sugito N (2009). Counterbalance between RB inactivation and miR-17-92 overexpression in reactive oxygen species and DNA damage induction in lung cancers. *Oncogene*.

[140] Wu X, Piper-Hunter MG, Crawford M (2009). MicroRNAs in the pathogenesis of lung cancer. *Journal of Thoracic Oncology*.

[141] Hu Z, Chen X, Zhao Y (2010). Serum microRNA signatures identified in a genome-wide serum microRNA expression profiling predict survival of non-small-cell lung cancer. *Journal of Clinical Oncology*.

[142] Lu Y, Govindan R, Wang L MicroRNA profiling and prediction of recurrence/relapse-free survival in stage I lung cancer.

[143] Wiggins JF, Ruffino L, Kelnar K (2010). Development of a lung cancer therapeutic based on the tumor suppressor microRNA-34. *Cancer Research*.

[144] Chen Y, Zhu X, Zhang X, Liu B, Huang L (2010). Nanoparticles modified with tumor-targeting scFv deliver siRNA and miRNA for cancer therapy. *Molecular Therapy*.

[145] Chen Z, Zeng H, Guo Y (2010). MiRNA-145 inhibits non-small cell lung cancer cell proliferation by targeting c-Myc. *Journal of Experimental and Clinical Cancer Research*.

[146] Muniyappa MK, Dowling P, Henry M (2009). MiRNA-29a regulates the expression of numerous proteins and reduces the invasiveness and proliferation of human carcinoma cell lines. *European Journal of Cancer*.

[147] Galluzzi L, Morselli E, Vitale I (2010). miR-181a and miR-630 regulate cisplatin-induced cancer cell death. *Cancer Research*.

[148] Cho WC (2010). MicroRNAs as therapeutic targets for lung cancer. *Zhongguo Fei Ai Za Zhi*.

[149] Bader AG, Brown D, Winkler M (2010). The promise of microRNA replacement therapy. *Cancer Research*.

[150] Nana SP, Croce CM (2011). MicroRNAs as therapeutic targets in cancer. *Translational Research*.

[151] Krützfeldt J, Rajewsky N, Braich R (2005). Silencing of microRNAs in vivo with ‘antagomirs’. *Nature*.

[152] Garzon R, Marcucci G, Croce CM (2010). Targeting microRNAs in cancer: rationale, strategies and challenges. *Nature Reviews Drug Discovery*.

[153] Elmén J, Lindow M, Silahtaroglu A (2008). Antagonism of microRNA-122 in mice by systemically administered LNA-antimiR leads to up-regulation of a large set of predicted target mRNAs in the liver. *Nucleic Acids Research*.

[154] Chi SW, Zang JB, Mele A, Darnell RB (2009). Argonaute HITS-CLIP decodes microRNA-mRNA interaction maps. *Nature*.

[155] Nunnari G, Schnell MJ (2011). MicroRNA-122: a therapeutic target for hepatitis C virus (HCV) infection. *Frontiers in Bioscience*.

[156] Thu KL, Chari R, Lockwood WW, Lam S, Lam WL (2011). miR-101 DNA copy loss is a prominent subtype specific event in lung cancer. *Journal of Thoracic Oncology*.

[157] Zhang JG, Guo JF, Liu DL, Liu Q, Wang JJ (2011). MicroRNA-101 exerts tumor-suppressive functions in non-small cell lung cancer through directly targeting enhancer of zeste homolog 2. *Journal of Thoracic Oncology*.

[158] Zou HY, Li Q, Lee JH (2007). An orally available small-molecule inhibitor of c-Met, PF-2341066, exhibits cytoreductive antitumor efficacy through antiproliferative and antiangiogenic mechanisms. *Cancer Research*.

[159] Ebert MS, Neilson JR, Sharp PA (2007). MicroRNA sponges: competitive inhibitors of small RNAs in mammalian cells. *Nature Methods*.

[160] Wang J, Liu X, Wu H (2010). CREB up-regulates long non-coding RNA, HULC expression through interaction with microRNA-372 in liver cancer. *Nucleic Acids Research*.

[161] Poliseno L, Salmena L, Zhang J, Carver B, Haveman WJ, Pandolfi PP (2010). A coding-independent function of gene and pseudogene mRNAs regulates tumour biology. *Nature*.

[162] Alimonti A, Carracedo A, Clohessy JG (2010). Subtle variations in Pten dose determine cancer susceptibility. *Nature Genetics*.

[163] Ghildiyal M, Zamore PD (2009). Small silencing RNAs: an expanding universe. *Nature Reviews Genetics*.

[164] Holley CL, Topkara VK (2011). An introduction to small non-coding RNAs: miRNA and snoRNA. *Cardiovascular Drugs and Therapy*.

[165] Phizicky EM, Hopper AK (2010). tRNA biology charges to the front. *Genes and Development*.

[166] Hannan KM, Hannan RD, Rothblum LI (1998). Transcription by RNA polymerase I. *Frontiers in Bioscience*.

[167] Taft RJ, Kaplan CD, Simons C, Mattick JS (2009). Evolution, biogenesis and function of promoter-associated RNAs. *Cell Cycle*.

[168] Scaruffi P (2011). The transcribed-ultraconserved regions: a novel class of long noncoding RNAs involved in cancer susceptibility. *TheScientificWorldJournal*.

[169] D’Errico I, Gadaleta G, Saccone C (2004). Pseudogenes in metazoa: origin and features. *Brief Funct Genomic Proteomic*.

[170] Wagner EG, Flärdh K (2002). Antisense RNAs everywhere?. *Trends in Genetics*.

[171] Wang ZX, Bian HB, Wang JR, Cheng ZX, Wang KM, De W (2011). Prognostic significance of serum miRNA-21 expression in human non-small cell lung cancer. *Journal of Surgical Oncology*.

[172] Kanzaki H, Ito S, Hanafusa H (2011). Identification of direct targets for the miR-17-92 cluster by proteomic analysis. *Proteomics*.

[173] Beane J, Vick J, Schembri F (2011). Characterizing the impact of smoking and lung cancer on the airway transcriptome using RNA-Seq. *Cancer Prevention Research*.

[174] Zhang JG, Wang JJ, Zhao F, Liu Q, Jiang K, Yang GH (2010). MicroRNA-21 (miR-21) represses tumor suppressor PTEN and promotes growth and invasion in non-small cell lung cancer (NSCLC). *Clinica Chimica Acta*.

[175] Sun Y, Bai Y, Zhang F, Wang Y, Guo Y, Guo L (2010). miR-126 inhibits non-small cell lung cancer cells proliferation by targeting EGFL7. *Biochemical and Biophysical Research Communications*.

[176] Kohda M, Hoshiya H, Katoh M (2001). Frequent loss of imprinting of *IGF2* and *MEST* in lung adenocarcinoma. *Molecular Carcinogenesis*.

[177] Zuker M, Stiegler P (1981). Optimal computer folding of large RNA sequences using thermodynamics and auxiliary information. *Nucleic Acids Research*.

[178] Rivas E, Eddy SR (1999). A dynamic programming algorithm for RNA structure prediction including pseudoknots. *Journal of Molecular Biology*.

[179] Reeder J, Giegerich R (2004). Design, implementation and evaluation of a practical pseudoknot folding algorithm based on thermodynamics. *BMC Bioinformatics*.

[180] Sczyrba A, Krüger J, Mersch H, Kurtz S, Giegerich R (2003). RNA-related tools on the Bielefeld Bioinformatics Server. *Nucleic Acids Research*.

[181] Nawrocki EP, Kolbe DL, Eddy SR (2009). Infernal 1.0: inference of RNA alignments. *Bioinformatics*.

[182] Washietl S, Hofacker IL, Stadler PF (2005). Fast and reliable prediction of noncoding RNAs. *Proceedings of the National Academy of Sciences of the United States of America*.

[183] Rivas E, Eddy SR (2001). Noncoding RNA gene detection using comparative sequence analysis. *BMC Bioinformatics*.

[184] Pedersen JS, Bejerano G, Siepel A (2006). Identification and classification of conserved RNA secondary structures in the human genome. *PLoS Computational Biology*.

[185] Harmanci AO, Sharma G, Mathews DH (2007). Efficient pairwise RNA structure prediction using probabilistic alignment constraints in Dynalign. *BMC Bioinformatics*.

[186] Gorodkin J, Heyer LJ, Stormo GD (1997). Finding the most significant common sequence and structure motifs in a set of RNA sequences. *Nucleic Acids Research*.

[187] Yao Z, Weinberg Z, Ruzzo WL (2006). CMfinder—a covariance model based RNA motif finding algorithm. *Bioinformatics*.

